# The weight loss grading system as a predictor of cancer cachexia in oesophageal cancer survivors

**DOI:** 10.1038/s41430-022-01183-6

**Published:** 2022-08-18

**Authors:** Poorna Anandavadivelan, Asif Johar, Pernilla Lagergren

**Affiliations:** 1 Surgical Care Sciences, Department of Molecular Medicine and Surgery, Karolinska Institutet, Stockholm, Sweden; 2 Department of Surgery and Cancer, Imperial College London, London, UK

**Keywords:** Oesophageal cancer, Digestive signs and symptoms

## Abstract

**Background:**

Oesophageal cancer survivorship is afflicted by cancer cachexia related weight loss and nutrition impact symptoms. Identifying the factors which predict cancer cachexia specifically is warranted in order to identify those at risk and render the right kind of support. We aimed to assess if preoperative and postoperative body mass index (BMI) adjusted weight loss grading system (WLGS) is predictive of cancer cachexia at one year after surgery for oesophageal cancer.

**Methods:**

Data were used from a prospective nationwide cohort study on patients operated on for oesophageal cancer in Sweden between 2013 and 2018 included at one year after surgery. The study exposure is BMI adjusted weight loss graded into one of five distinct weight loss grades (grades 0–4), defined in accordance with the WLGS by combining BMI and percentage weight loss, assessed at two clinical time points: preoperative and at 6 months post-surgery for oesophageal cancer. The study outcome is subjective measures of cancer cachexia one year after surgery, assessed using the cancer-cachexia specific questionnaire EORTC QLQ-CAX24. Multivariable linear regression models calculated mean score differences (MD) with 95% confidence intervals (CI) adjusted for predefined confounders. Statistical significance at *p* < 0.05 together with a clinically relevant difference of 10-points in mean scores was considered as a significant difference.

**Results:**

Among a total of 232 patients, the highest grade of preoperative WLGS 4 was associated with significantly worse physical decline than lower grades of WLGS 1 (MD -10, 95% CI: −20 to −1) and WLGS 2 (MD −11, 95% CI: −20 to −2). Those with preoperative WLGS 2, 3 and 4 reported lower scores on the adequacy of information on weight loss provided to them than those with preoperative WLGS 0. Those with the highest postoperative WLGS 4 had greater eating and weight loss worry than WLGS 2 (MD −17, 95% CI: −32 to −3) and WLGS 3 (MD −11, 95% CI: −21 to −2) and worse physical decline than WLGS 0 (MD −14, 95% CI: −25 to −2).

**Conclusions:**

Higher grades of both preoperative and postoperative WLGS are predictive of cancer cachexia related physical decline one year after surgery for oesophageal cancer. Additionally, preoperative and postoperative WLGS were also predictive of inadequate information concerning weight loss and more worry regarding eating and weight loss, respectively. The WLGS may be an effective risk prediction tool for postoperative cachexia related physical decline in patients undergoing treatment for oesophageal cancer emphasizing its usability in the clinical setting.

## Introduction

Despite improving survival rates with multimodal treatments such as chemotherapy and radiotherapy, an extensive surgical procedure remains the cornerstone of curatively intended treatment for oesophageal cancer [1, 2]. Cancer cachexia is a multifactorial syndrome characterised by the loss of muscle mass with or without loss of fat mass. Patients with oesophageal cancer face significant weight loss at diagnosis from cancer-induced systemic inflammation (primary cachexia) and also from swallowing difficulties due to tumour obstruction in the oesophagus (secondary cachexia/secondary nutritional impact symptoms). Moreover, neoadjuvant therapy followed by extensive surgery, which is the curative treatment for oesophageal cancer, contributes to loss of weight during treatment. Post-operative recovery is long (on an average one year) [1] and patients are faced with challenges that hinder the recovery with respect to nutritional status with prevalent weight loss more prominent in those with a higher pre-operative body mass index (BMI) [3–5]. The use of minimum reported degrees of weight loss is heterogeneous, not based on specific values that relate to an adverse clinical outcome and not always evaluated based on the context of initial body reserves. This is more imperative in the context of oesophageal cancer since most patients are overweight/obese at diagnosis with BMI being a recognized risk factor for the histological sub-type, adenocarcinoma [6]. The weight loss grading system (WLGS) addresses these shortcomings in redefining clinically important weight loss and is included in the current international clinical practice guidelines for nutrition and cancer [7, 8]. Previous evidence indicates that higher preoperative BMI is a prognostic marker of greater postoperative weight loss after oesophageal cancer surgery. Although after surgery, the primary cachexia from the tumour-induced systemic inflammation is not present, patients are challenged from secondary cachexia or nutrition impact symptoms that may lead to progression of the primary cachexia as post-surgical weight loss and functional deterioration. There is a crucial need to investigate the implementation of the WLGS in patients with oesophageal cancer to determine its prognostic value and its association with long term cancer cachexia. The aim of the present study is to evaluate if BMI adjusted weight loss according to the WLGS assessed both before and at 6 months after surgery for oesophageal cancer is a predictor of long-term cancer cachexia at one year after surgery. We hypothesized that a higher grade of BMI adjusted weight loss before surgery and at 6 months following surgery according to the WLGS would have a negative impact on cancer cachexia specific aspects of health-related quality of life (HRQL) i.e., food aversion, eating difficulties, loss of control, and physical decline, at one year after surgery for oesophageal cancer.

## Methods

### Data source and study design

Data for this study are obtained from the *Oesophageal Surgery in Cancer patients: Adaptation and Recovery (OSCAR)* cohort, a nationwide and prospective study of patients who were operated on for oesophageal cancer in Sweden from 2013. The data collection for the OSCAR study is ongoing. The OSCAR study is explained in detail elsewhere [9]. Briefly, patients were identified from pathology units at the eight hospitals treating oesophageal cancer in Sweden and were invited to participate in the cohort study one year after surgery (i.e., from 2014). Follow-ups were conducted at regular intervals until five years postoperatively. The OSCAR study was approved by the Regional Ethical Review Board in Stockholm, Sweden (diary number 2013/844-31/1) and all participants provided written informed consent.

### Data collection

A wide range of patient-reported outcome measures were collected in the OSCAR study at the one year-assessment by the research nurse who conducted a home visit to the patients via a combination of interview questions and validated written self-report questionnaires. For the purpose of the present study, patient-reported cancer-cachexia assessed with a validated questionnaire at the one-year assessment was included. A systematic data collection from a review of medical records were conducted by a group of researchers and clinicians to obtain detailed clinical data according to a predefined study protocol to ensure uniformity of the data collection. For this study, patient characteristics including age, sex, preoperative BMI, preoperative and postoperative weight loss and co-morbidity severity, according to the Charlson co-morbidity score [10], were included. Tumour characteristics included tumour histology and tumour stage, classified according to the Union for International Cancer Control [11]. Treatment-related characteristics were neoadjuvant therapy, type of operation and postoperative complications, classified according to the Clavien-Dindo classification system [12]. Also, the extent of enteral and/or parenteral nutrition support was used.

### Study measures

**Fig. 1:**
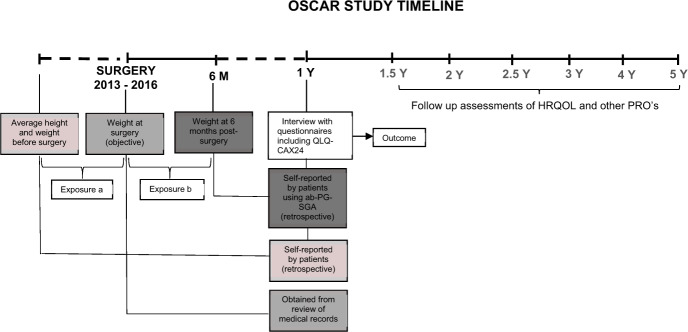
Timeline of the OSCAR study data collection. Data assessments points for the exposure of **a** pre-operative weight loss grading system and **b** post-operative weight loss grading system and the outcome QLQ-CAX24 are indicated. HRQOL Health-related quality of life, QLQ-CAX24 Cancer-cachexia specific questionnaire, PRO’s Patient reported outcomes, ab-PG-SGA Abridged patient generated subjective global assessment.

The study exposure is BMI adjusted weight loss graded into one of five distinct weight loss grades (grades 0–4), defined in accordance with the WLGS by combining BMI and percentage weight loss, assessed at two clinical time points: preoperative and post-operative at six months from the time of surgery for oesophageal cancer. To derive the two exposure groups, firstly the pre- and post-operative BMI and percentage weight loss were calculated (Table S1) and then graded from 0–4 as per the WLGS (Table S2). The timing of assessments of the pre- and post-operative exposure data from the OSCAR study timeline is outlined in Fig. 1.

a. Preoperative WLGS: Pre-operative BMI and percentage weight loss were calculated using the formula as shown in Table S1. For calculating the pre-operative BMI and percentage weight loss, weight at surgery (current weight) and the average weight and height of patients as an adult (previous weight) was used (Table S1). The data concerning weight at the time of operation were collected from medical records, and the data for average weight and height as an adult was reported by the patients themselves at the one-year follow up of the OSCAR study [Fig. 1].

b. Post-operative WLGS: Post-operative BMI and percentage weight loss were calculated using the formula as shown in Table S1. For calculating the post-operative BMI and percentage weight loss, weight at six months after surgery (current weight) and weight at surgery (previous weight) was used (Table S1). The data concerning weight at six months after surgery were reported by the patients using the nutritional assessment tool, abridged patient-generated subjective global assessment (ab-PG-SGA), also known as the PG-SGA short form at the one year follow up of the OSCAR study [Fig. 1]. The ab-PG-SGA includes the components: weight history, food intake, nutrition impact symptoms, and activities and functions. Patients were asked to report what their weight was six months retrospectively in the weight history component of ab-PG-SGA which was used in the present study. The ab-PG-SGA has been validated for use in oncology settings previously [13]. Weight at the time of surgery was obtained from medical records [Fig. 1].

The study outcome is subjective measures of cancer cachexia one year after surgery, assessed using the EORTC QLQ-CAX24 module, a cancer-cachexia specific questionnaire [Fig. 1] [14]. The questionnaire contains five multi-item scales (food aversion, eating and weight-loss worry, eating difficulties, loss of control, and physical decline) and four single items, thus comprising 24 items in total [14]. The questionnaire has four response alternatives: ‘not at all’, ‘a little’, ‘quite a bit’ and ‘very much’. Worse symptoms were considered present if the patients reported ‘quite a bit’ or ‘very much’ to any item within a scale or to a single item. Lesser symptoms were considered present if the patients reported ‘not at all’ or ‘a little’ to all items within a scale or to a single item. All scales and items were evaluated as specific outcomes. The scores were transformed into a scale ranging from 0 to 100 where for all the five scales and three of the four single items, a high mean score (MS) is indicative of greater problems. The exception is the single item on information about weight loss, ‘Has the information you have been given about your weight loss been adequate?’ a response of ‘not at all’ corresponded to inadequate provision of information and a high score indicated that adequate information has been given about weight loss [14]. The questionnaire was pilot tested in nine different European countries and the results of a validation study in 14 countries across the world with 557 participants are underway [15].

### Statistical analysis

Descriptive statistics were presented as counts (*n*) and proportions (%). Multivariable linear regression models were used to calculate the association between preoperative and postoperative WLGS and the cachexia scale scores from QLQ-CAX24. A linear model was used since a pre-established cut-off is not available for cachexia scores and mean scores difference (MD) of 10 or more points (10%) were tested for statistical significance. A 10-point difference was regarded as clinically relevant based on previous research [16] although no specific level of clinical relevance for the QLQ-CAX24 is available. With MD of at-least 10 and SD <= between the two groups, 5% level of significance and a two-sided test, the study has the power of 80%.

The preoperative WLGS analysis was adjusted for the following predefined confounders: sex (male/female), age at operation (continuous), comorbidities (Charlson comorbidity score 0/1/≥2), histological sub-type (adenocarcinoma and high-grade dysplasia/squamous cell carcinoma), pathological tumour stage (0-I/II/III-IV) and neoadjuvant therapy (yes/no). For the postoperative WLGS at six months after surgery in addition to the above confounders, type of operation (minimally invasive/hybrid thora/laparoscopic/open oesophagectomy), postoperative complications (Clavien-Dindo score (CDS); low grade (0-II)/high grade (III-IV), enteral and/or parenteral nutrition support for at least 1 week postoperatively (yes/no) was adjusted for. Statistical analysis was conducted using SAS 9.4 software (SAS Institute, Cary, NC).

## Results

### Response rate and patient characteristics

Between January 2013 and 15 May 2019, a total of 839 patients underwent surgery for cancer of the oesophagus in Sweden. Of those, 636 patients were alive at the one-year follow-up, 113 were unreachable and one patient was excluded owing to cognitive impairment, leaving a total of 522 patients eligible for inclusion in the OSCAR cohort and 438 eligible for the current study as 84 were included in the cohort before the EORTC QLQ-CAX24 questionnaire was introduced. Among those identified as eligible, 173 (39%) declined to participate for reasons namely, cancer recurrence (*n* = 25) or other reasons (because they did not want/had the energy to participate (*n* = 119), were too sick (*n* = 28), other reasons (*n* = 1)). In total, 253 (58% of eligible) completed the EORTC QLQ-CAX24 questionnaire, and 232 had complete data concerning WLGS before operation and clinical data from medical charts, hence were included in the analysis.

Socio-demographic and clinical characteristics of the patients included in the present study is provided in Table 1 and no statistical test were performed to compare these characteristics. Briefly, the majority of patients were men (92.7%), with a mean age of 67 years. The majority of patients had adenocarcinoma or high-grade dysplasia (83.6%), around 79.3% received neoadjuvant therapy before surgery. High grade postoperative complications were developed in 35% of patients and 82% received enteral/parenteral nutrition support.

**Table 1:** Patient and clinical characteristics of patients operated for oesophageal cancer (*n* = 232) and categorized by preoperative weight loss grading system.

Characteristics	Overall (*n* = 232)	Pre-operative weight loss grading system				
		0 (*n* = 80)	1 (*n* = 33)	2 (*n* = 44)	3 (*n* = 52)	4 (*n* = 23)
Sex						
Male	215 (92.7)	75 (93.7)	28 (85.0)	42 (95.4)	49 (94.2)	21 (91.3)
Female	17 (7.3)	5 (6.3)	5 (15.0)	2 (4.6)	3 (5.8)	2 (8.7)
Age at surgery*	67 (66–68)	67 (65–69)	68 (64–71)	67 (65–70)	67 (65–69)	69 (66-72)
Charlson co-morbidity score						
0	100 (43.1)	32 (40.0)	13 (39.4)	23 (52.3)	23 (44.2)	9 (39.1)
1	77 (33.2)	23 (28.7)	15 (45.5)	12 (27.3)	17 (32.7)	10 (43.5)
≥2	55 (23.7)	25 (31.3)	5 (15.1)	9 (20.4)	12 (23.1)	4 (17.4)
Histological sub-type						
Adenocarcinoma and HGD	194 (83.6)	72 (90.0)	27 (81.8)	35 (79.5)	44 (84.6)	16 (69.6)
Squamous cell carcinoma	38 (16.4)	8 (10.0)	6 (18.2)	9 (20.5)	8 (15.4)	7 (30.4)
Postoperative tumour stage						
I	80 (34.5)	34 (42.5)	12 (36.4)	11 (25.0)	19 (36.5)	4 (17.4)
II	74 (31.9	21 (26.3)	11 (33.3)	16 (36.4)	17 (32.7)	9 (39.1)
III-IV	78 (33.6)	25 (31.2)	10 (30.3)	17 (38.6)	16 (30.8)	10 (43.5)
Neo-adjuvant therapy						
Yes	184 (79.3)	61 (76.3)	22 (66.7)	39 (88.6)	45 (86.5)	17 (73.9)
No	48 (20.7)	19 (23.7)	11 (33.3)	5 (11.4)	7 (13.5)	6 (26.1)
Type of operation						
Minimally Invasive	95 (41.0)	25 (31.3)	11 (33.3)	21 (47.7)	27 (51.9)	11 (47.8)
Hybrid Thora/Laparoscopic	85 (36.6)	33 (41.2)	14 (42.4)	15 (34.1)	15 (28.9)	8 (34.8)
Open Oesophagectomy	52 (22.4)	22 (27.5)	8 (24.2)	8 (18.2)	10 (19.2)	4 (17.4)
Post-operative complications						
Low grade (CDS 0-II)	150 (64.7)	54 (67.5)	21 (63.6)	24 (54.6)	38 (73.1)	13 (56.5)
High grade (CDS III-IV)	82 (35.3)	26 (32.5)	12 (36.4)	20 (45.4)	14 (26.9)	10 (43.5)
Enteral/Parenteral nutrition support						
Yes	190 (81.9)	62 (77.5)	29 (87.9)	37 (84.1)	41 (78.8)	21 (91.3)
No	42 (18.1)	18 (22.5)	4 (12.1)	7 (15.9)	11 (21.2)	2 (8.7)

Descriptive statistics presented as counts (*n*) and proportions (%). Values are number of patients and percentages within brackets unless specified; *Average age in years (95% confidence interval).

*HGD* High grade dysplasia, *CDS* Clavien Dindo Score.

### Preoperative and postoperative weight loss grades

**Fig. 2:**
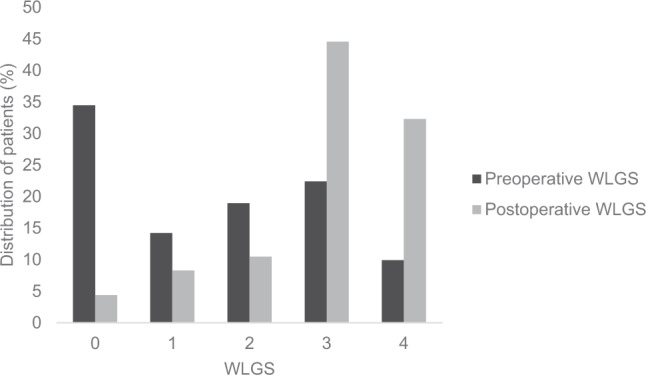
Distribution of patients as per pre- and post-operative weight loss grading system (WLGS) (0–4). Descriptive statistics presented as proportion (%) of patients in the exposure groups of pre- and post-operative weight loss grading system.

The proportion of patients with weight loss grades 0–4 before surgery compared to after surgery are represented in Fig. 2. The preoperative weight loss grades categorized as per the WLGS showed that from the 232 patients included, WLGS 0 had the largest proportion (34.5%), followed by WLGS 3 (22.4%), WLGS 2 (19%), WLGS 1 (14.2%), and the smallest proportion were in the highest grade of weight loss, WLGS 4 (9.9%) [Fig. 2]. Whilst, according to the WLGS assessed at six months after surgery, WLGS 3 (44.5%) had the highest proportion, followed by WLGS 4 (32.3%), WLGS 2 (10.5%), WLGS 1 (8.3%), and the lowest proportion were in the lowest grade of WLGS 0 (4.4%) [Fig. 2].

The clinical characteristics among the weight loss grades indicated that more patients with WLGS 0 (31.3%) had a higher comorbidity score than those in the other categories of WLGS 1–4 (15.1% to 23.1%). More patients (43.5%) with WLGS 4 had a higher pathological tumour stage (III-IV) than those with the least WLGS 0 (31%). As many as 91.3% of patients in the WLGS 4 received enteral or parenteral nutrition support compared to 77.5% in the WLGS 1 (Table 1). More patients (80%) with WLGS 4 at six months after surgery had received neoadjuvant therapy compared to 60% in WLGS 0 (data not shown). More patients (47%) with WLGS 1 at six months after surgery had higher co-morbidities than those in other WLGS (0, 2–4) ranging from 17.6% to 25% (data not shown).

### Preoperative weight loss grades and cancer cachexia scores

**Fig. 3:**
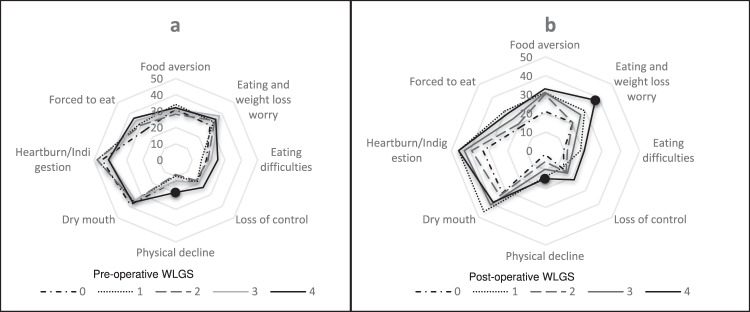
Adjusted mean scores on the EORTC QLQ-CAX24 (outcome) for the two exposure groups. Exposure groups are **a** pre-operative weight loss grading system (WLGS) and **b** post-operative weight loss grading system. Multivariable linear regression models were used to calculate the association between pre- and post-operative WLGS and the scores from EORTC QLQ-CAX24 and adjusted for predefined confounders. Circles indicate clinically relevant MS ( ≥10 points) and statistically significant differences as per the multivariable linear regression models (*p* < 0.050) between a pre-operative WLGS 1–4 and 2–4 in the physical decline scale, b post-operative WLGS 2–4 and 3–4 in the eating and weight loss worry scale and WLGS 0−4 in the physical decline scale. MS Mean scores, EORTC European Organisation for Research and Treatment of Cancer, QLQ-CAX24 Cancer-cachexia specific questionnaire, WLGS Weight loss grading system.

**Fig. 4:**
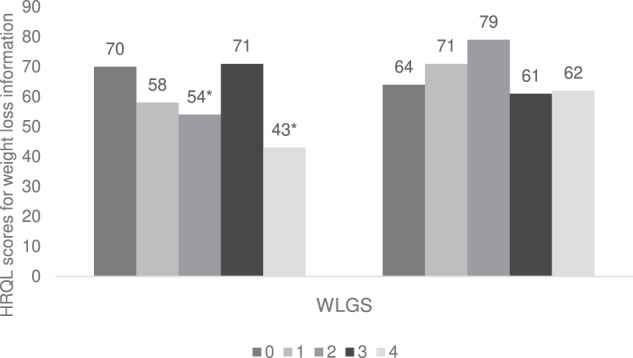
Adjusted mean scores for the single item (weight loss information) in EORTC QLQ-CAX24 (outcome) for the two exposure groups. Exposure groups are **a** pre-operative weight loss grading system (WLGS) and **b** post-operative weight loss grading system. Multivariable linear regression models were used to calculate the association between pre- and post-operative WLGS and the scores for the single item, ‘weight loss information’ from the EORTC QLQ-CAX24 and adjusted for predefined confounders. *Clinically relevant MS ( ≥10 points) and statistically significant differences as per the multivariable linear regression models (*p* < 0.050) between pre-operative WLGS 2–3, 0–2 and 0–4 in the single item information on weight loss. MS Mean scores, EORTC European Organisation for Research and Treatment of Cancer, QLQ-CAX24 Cancer-cachexia specific questionnaire, WLG Weight loss grading system.

Among the five dimensions of the QLQ-CAX24 questionnaire, the highest mean scores (MS) from the five weight loss grades were reported in the eating and weight loss worry dimension (MS 30 to 38) and food aversion dimension (MS 28 to 34) [Table 2]. Conversely, the lowest scores were reported in the physical decline dimension (MS 9 to 20). Among the four single items, the highest MS were received for heartburn/indigestion (MS 41 to 48) followed by dry mouth (MS 35 to 39) [Table 2]. The multivariable analysis of the cancer cachexia scores among the five grades of weight loss indicated that significant differences with clinical relevance were observed in the physical decline dimension and the single item, information on weight loss (Figs. 3, 4). The MD in the physical decline dimension were clinically relevant and statistically significant between WLGS 1 and WLGS 4 (MD −10, 95% Confidence Interval [CI] −20 to −1), and WLGS 2 and WLGS 4 (MD −11, 95% CI: −20 to −2) [Fig. 3]. Likewise, in the single item information on weight loss, clinically relevant and statistically significant differences in MS were observed between WLGS 2 and WLGS 3 (MD −18, 95% CI: −33 to −3), WLGS 0 and WLGS 2 (MD 16, 95% CI: 2 to 30), and WLGS 0 and WLGS 4 (MD 26, 95% CI: 8 to 44) [Fig. 4]. In none of the other dimensions and single items were significant differences observed in the cachexia scores between the five grades of weight loss [Table 2, Fig. 3].

**Table 2:** Adjusted mean scores on the EORTC QLQ-CAX24 (outcome) reported by patients at one year after surgery for oesophageal cancer for the pre-operative weight loss grading system 0–4 (exposure).

QLQ-CAX24 Dimensions	Pre-operative weight loss grading system				
	0	1	2	3	4
Mean Scores (95% Confidence Interval)					
Food aversion	31 (24 to 39)	34 (25 to 43)	28 (19 to 37)	31 (22 to 39)	32 (22 to 43)
Eating and weight loss worry	30 (19 to 40)	33 (21 to 45)	34 (21 to 46)	38 (26 to 49)	35 (20 to 49)
Eating difficulties	19 (12 to 26)	16 (8 to 25)	20 (11 to 28)	22 (14 to 31)	26 (15 to 36)
Loss of control	19 (13 to 26)	19 (11 to 27)	18 (10 to 26)	20 (12 to 27)	24 (14 to 33)
Physical decline	13 (7 to 19)	10 (3 to 17)	9 (2 to 16)	13 (6 to 20)	20 (12 to 29)^*^
Dry mouth	39 (29 to 49)	36 (24 to 48)	36 (23 to 48)	35 (23 to 46)	37 (22 to 51)
Heartburn/Indigestion	45 (34 to 55)	48 (35 to 60)	48 (35 to 61)	41 (29 to 54)	41 (26 to 57)
Forced to eat	26 (16 to 36)	31 (19 to 43)	30 (17 to 42)	34 (22 to 45)	36 (22 to 51)
Weight loss information	70 (57 to 82)	58 (43 to 73)	54 (38 to 69)*	71 (57 to 86)	43 (25 to 61)^*^

Multivariable linear regression models were used to calculate the association between pre-operative WLGS and the scores from EORTC QLQ-CAX24 and adjusted for predefined confounders.

*MS* Mean Scores, *EORTC* European Organisation for Research and Treatment of Cancer, *QLQ-CAX24* Cancer-cachexia specific questionnaire, *WLGS* Weight loss grading system.

*Clinically relevant MS ( ≥10 points) and statistically significant differences as per the multivariable linear regression models (*p* < 0.050) between pre-operative WLGS 1–4 and 2–4 in the physical decline scale and WLGS 2–3, 0–2 and 0–4 in the single item information on weight loss.

### Postoperative weight loss grades and cancer cachexia scores

Among the dimensions of the QLQ-CAX24 as per the five grades of postoperative WLGS at six months after surgery, the highest MS were reported in the eating and weight loss worry dimension (MS 21 to 38) and food aversion dimension (MS 21 to 33) [Table 3]. Whereas, the lowest scores were reported in the physical decline dimension (MS 2 to 15). Likewise, among single items, the highest MS were received for heartburn/indigestion (MS 33 to 46) and dry mouth (MS 32 to 46) [Table 3]. In almost all the dimensions of QLQ-CAX24 except for the loss of control and in all the four single items, clinically relevant differences were seen between at least two or more WLGS groups, however, these did not reach statistical significance (Table 3, Figs. 3, 4). The linear regression model between the exposure of WLGS at months after surgery and the outcome of cachexia scores showed MD with clinical relevance and statistical significance in the dimensions of eating and weight loss worry and physical decline (Figs. 3, 4). In the eating and weight loss worry dimension, clinically relevant and statistically significant differences were noticed in MS between WLGS 2 and WLGS 4 (MD −17, 95% CI: −32 to −3), and WLGS 3 and WLGS 4 (MD −11, 95% CI: −21 to −2) [Fig. 3]. Further, in the physical decline dimension, MS differed statistically significantly between WLGS 0 and WLGS 4 (MD −14, 95% CI: −25 to −2) [Fig. 3].

**Table 3:** Adjusted mean scores on the EORTC QLQ-CAX24 (outcome) reported by patients at one year after surgery for oesophageal cancer for the post-operative weight loss grading system (exposure).

QLQ-CAX24 Dimensions	Post-operative weight loss grading system				
	0	1	2	3	4
Mean Scores (95% Confidence Interval)					
Food aversion	21 (5 to 37)	31 (20 to 43)	30 (18 to 43)	31 (23 to 40)	33 (25 to 41)
Eating and weight loss worry	21 (0 to 43)	30 (15 to 46)	21 (5 to 38)	27 (16 to 38)	38 (27 to 49)^*^
Eating difficulties	10 (−6 to 25)	19 (8 to 30)	12 (0 to 23)	15 (7 to 23)	21 (13 to 29)
Loss of control	14 (0 to 28)	13 (3 to 23)	16 (5 to 27)	17 (9 to 24)	22 (15 to 29)
Physical decline	2 (−11 to 14)	14 (5 to 23)	6 (−4 to 16)	10 (3 to 16)	15 (9 to 22)
Dry mouth	32 (10 to 54)	46 (30 to 61)	34 (17 to 50)	41 (29 to 52)	39 (28 to 50)
Heartburn/Indigestion	33 (10 to 56)	46 (30 to 63)	39 (22 to 57)	43 (31 to 55)	46 (34 to 57)
Forced to eat	18 (−3 to 39)	30 (15 to 46)	20 (3 to 36)	24 (13 to 35)	28 (18 to 39)
Weight loss information	64 (35 to 92)	71 (51 to 91)	79 (58 to 101)	61 (47 to 76)	62 (48 to 76)^*^

Multivariable linear regression models were used to calculate the association between post-operative WLGS and the scores from EORTC QLQ-CAX24 and adjusted for predefined confounders.

*MS* Mean Scores, *EORTC* European Organisation for Research and Treatment of Cancer, *QLQ-CAX24* Cancer-cachexia specific questionnaire, *WLGS* Weight loss grading system.

*Clinically relevant MS ( ≥10 points) and statistically significant differences as per the multivariable linear regression models (*p* < 0.050) between post-operative WLGS 2–4 and 3–4 in the eating and weight loss worry scale and WLGS 0–4 in the physical decline scale.

## Discussion

This population-based prospective cohort study on patients who underwent surgery for oesophageal cancer, exploring if the WLGS is a predictor of long-term cancer cachexia indicated that higher grades of both preoperative and postoperative WLGS are associated with cancer cachexia related physical decline at one year after surgery for oesophageal cancer. Additionally, higher grades of preoperative WLGS were also associated with inadequate information on weight loss aspects of cancer cachexia. Meanwhile, higher grades of postoperative WLGS was also predictive of eating and weight loss worry among patients. The results signify that the WLGS maybe a useful predictor of specific cancer cachexia related decline in oesophageal cancer patients.

Several methodological aspects in the present study add to its strength. The population-based nationwide data collection with a longitudinal design minimised selection bias and increased generalisability of the results. The OSCAR cohort is comprised mostly of male patients, which is typical for oeophageal cancer and hence considered representative of the disease demographics and generalisable. Adherence to a strict protocol for data collection further reduced the scope for bias, and another added strength is the unique home-based data collection which reduced missing values. However, selection bias through non-participation of those who were unreachable and declined participation in the study cannot be neglected and may be considered a potential limitation. The choice of the WLGS as the assessment tool for the exposure and EORTC QLQ-CAX24 for the assessment of the outcome is also a strength in the present study. The WLGS has marked a crucial step in redefining clinically important weight loss where BMI is included and has thus been included in the current international clinical practice guidelines for nutrition and cancer [8]. Moreover, the WLGS has also been shown to be associated with cachexia-related domains such as reduced dietary intake, anorexia, reduced performance steps, and increased fatigue, suggesting that the WLGS may be useful in cachexia classification [17]. However, the effectiveness of the robust WLGS in differentiating groups of patients with worse cachexia in patients who have undergone treatment for oesophageal cancer, a patient group highly vulnerable to weight loss, has not been studied previously and this is the first study to test these outcomes. The WLGS may prove to be a standardized weight loss model since it includes BMI, which is an important determinant for prognosis for this patient group. The WLGS is a recommended model for weight loss in cancer patients in general, but this study adds to the significance of how it relates specifically to oesophageal cancer survivors. One limitation may have been that the QLQ-CAX24 questionnaire was developed for use in patients with cancer cachexia in whom the metabolic component of primary cachexia from the tumour-induced systemic inflammation is present. We have used the questionnaire one year after surgery for the cancer when this is no longer an issue, however, the persisting problems of lingering secondary cachexia and nutrition impact symptoms owing to the extensive surgery itself, substantiates the use of the questionnaire in this survivor group. Although the mechanisms leading to cachexia are different in the above two groups, the dimensions and single items covered by the tool are very much pertinent issues even at one year after undergoing treatment for oesophageal cancer. Another limitation may have been lower statistical power in the various WLGS as implied by the wide confidence intervals in the results of the linear regression.

In our study, both the higher grades of preoperative and postoperative WLGS was predictive of physical decline at one year after surgery for oesophageal cancer. In the treatment of patients with oesophageal cancer, resection surgery remains the cornerstone of curatively intended treatment [1]. The surgery by itself is extraordinarily extensive and the post-operative recovery is long (on an average one year) with deterioration in several aspects of health-related quality of life [1]. Many patients face several post-operative complications [18] and are also faced with challenges of decreased muscle strength and cardiorespiratory fitness which are key determinants of physical function defined as an individual’s capacity to undertake tasks that are crucial to daily living [19]. In patients undergoing neoadjuvant therapy for oesophageal cancer, significant reductions in physical function is observed [19]. Similarly, physical function and habitual physical activity levels are compromised significantly post-resection [19, 20]. Consequently, there is clear justification for the early identification of patients who are at risk for such physical decline in order to facilitate the early introduction of measures to counteract the impairment in physical function such as prehabilitation and rehabilitation before and after surgery, respectively. Moreover, higher grades of the preoperative WLGS were also associated with lower adequacy of information regarding weight loss that the patients received, indicating that those who had faced a higher grade of weight loss reported not receiving adequate information regarding weight loss. These results indicate the importance of the need to better inform patients who are facing higher weight loss in healthcare. This may be in the form of more elaborate and frequent counselling sessions with these patients. The results of the present study also showed that higher postoperative grades of the WLGS were associated with greater worry regarding eating and weight loss. These results convey that the patients facing a greater degree of weight loss, worry more regarding the eating difficulties and the weight loss they are faced with after the surgery. The psychosocial concerns regarding eating problems and weight loss are central issues for those with higher weight loss and in turn, stresses the need for heightened psychological support in this patient sub-group long-term after surgery.

Our results highlight that the WLGS may be a good risk prediction model for the cancer cachexia related physical decline in patients undergoing treatment with oesophageal cancer emphasizing its usability in the clinical setting. The persisting and long-term issues concerning eating and weight status are also a major challenge for health care and the WLGS is a more clinically relevant and robust tool for cachexia related physical decline after surgery for oesophageal cancer.

## Supplementary information

Table S1

Table S2

## Data Availability

The datasets generated and/or analyzed in the current study will not be publicly available due to the ethical review act but will be available from the principal investigators of the study on reasonable request.
