# Supplementary material for: The weight loss grading system as a predictor of cancer cachexia in oesophageal cancer survivors
Source: Eur J Clin Nutr. 2022 Aug 18;76(12):1755–61. doi: 10.1038/s41430-022-01183-6 (PMC9708569; doi:10.1038/s41430-022-01183-6)
Supplement: Supplementary file 1 — Table S1 [file 41430_2022_1183_MOESM1_ESM.docx]

**Table S1**. Calculations of body mass index and percentage weight loss at pre- and post-operative assessment of the weight loss grading system

| Clinical time-points of WLGS assessment  (exposure) | Body mass index  *[current weight (kg)/height (m2)]* | Percentage weight loss  *[(current weight (kg) – previous weight (kg))/previous weight(kg)]X100* |
| --- | --- | --- |
| Pre-operative | weight at surgery/height | [(weight at surgery - average weight as an adult)/average weight as an adult)]X100 |
| Post-operative | weight at 6 months after surgery/height | [(weight at 6 months after surgery –  weight at surgery/weight at surgery)]X100 |

**Supplementary table 1 legend**

*WLGS – Weight loss grading system; kg – Kilogram; m2- Square meter*

Weight at the time of operation were collected from medical records, average weight and height as an adult was reported by the patients themselves at the one-year follow up of the OSCAR study, weight at 6 months after surgery were reported by the patients using the nutritional assessment tool, abridged patient-generated subjective global assessment (ab-PG-SGA).
