# Supplementary material for: The weight loss grading system as a predictor of cancer cachexia in oesophageal cancer survivors
Source: Eur J Clin Nutr. 2022 Aug 18;76(12):1755–61. doi: 10.1038/s41430-022-01183-6 (PMC9708569; doi:10.1038/s41430-022-01183-6)
Supplement: Supplementary file 2 — Table S2 [file 41430_2022_1183_MOESM2_ESM.docx]

**Table S2.** Body mass index adjusted weight loss graded into one of five distinct weight loss grades (grades 0–4), defined in accordance with the weight loss grading system (WLGS) by combining body mass index and percentage weight loss

| Percentage weight loss (%) | Body mass index (kg/m2) | | | | |
| --- | --- | --- | --- | --- | --- |
|  | **≥28** | **25–27.9** | **22–24.9** | **20–21.9** | **<20** |
| < 2.5 | 0 | 0 | 1 | 1 | 3 |
| 2.5–5.9 | 1 | 2 | 2 | 2 | 3 |
| 6–10.9 | 2 | 3 | 3 | 3 | 4 |
| 11–14.9 | 3 | 3 | 3 | 4 | 4 |
| ≥ 15 | 3 | 4 | 4 | 4 | 4 |

**Supplementary table 2 legend**

*kg – Kilogram; m2- Square meter*
